# Hyperammonemia in Inherited Metabolic Diseases: A Case Report

**DOI:** 10.7759/cureus.94568

**Published:** 2025-10-14

**Authors:** Natalia Frankevich, Mzia Makieva, Olga Mikhailova, Vladimir Frankevich

**Affiliations:** 1 Obstetrics and Gynecology, National Medical Research Center for Obstetrics Gynecology and Perinatology Named After Academician V.I. Kulakov, Ministry of Healthcare of Russian Federation, Moscow, RUS

**Keywords:** ammonia, hyperammonemia, organic acidemias, ornithine trans-carbamylase deficiency, urea cycle disorders

## Abstract

Neonatal hyperammonemia is a medical emergency where diagnostic delay can lead to catastrophic neurological outcomes. This case report of a full-term male infant with ornithine transcarbamylase (OTC) deficiency highlights the critical challenges in its early recognition and management. The initial presentation of inborn errors of metabolism is often nonspecific in the neonatal period, and while biochemical testing is essential, it is not always rapidly available. Our experience underscores that a high index of clinical suspicion is paramount. Furthermore, this case reinforces the urgent need to expand newborn screening programs to facilitate a prompt diagnosis, allowing for the immediate initiation of treatment. Such measures are vital to prevent the irreversible pathological processes that lead to severe disability or increased mortality in these patients.

## Introduction

A number of congenital and acquired disorders can lead to hyperammonemia - an elevated level of ammonia in the blood. Ammonia is toxic to brain astrocytes, causing severe damage to the central nervous system (CNS). This manifests as life-threatening neuropsychiatric symptoms, the severity of which depends on the ammonia level, the duration of exposure, and brain maturity [1-3]. The primary cause of hyperammonemia in newborns is inherited metabolic diseases, such as urea cycle disorders (UCDs), organic acidemias, and fatty acid oxidation disorders. The most severe and frequent cases are observed with the first two types of pathologies [4,5].

The challenge in diagnosing inherited metabolic disorders in newborns lies in the fact that nearly 50% of cases present with non-specific symptoms that can be easily mistaken for manifestations of more common conditions. Frequently, in remote regions, early diagnosis cannot be achieved in a timely manner, which leads to increased rates of disability and infant mortality [6,7].

The most significant clinical manifestation of UCDs is hyperammonemic crisis. UCDs are caused by mutations in the genes encoding the six enzymes or two transporters that facilitate the urea cycle - the primary pathway for ammonia detoxification in the liver and, to a lesser extent, in the kidneys [2].

There are two main clinical presentations. The early-onset form (in neonates) is characterized by a near-complete absence of enzyme activity, leading to the rapid development of toxic encephalopathy proportional to the ammonia level. The late-onset form exhibits residual enzyme activity, resulting in a milder disease course.

In newborns, the crisis typically develops after the first 24 hours of life, triggered by catabolic stress, protein load, or medication use. The consequences of hyperammonemia include persistent impairments in cognitive and behavioral development [2,6].

Treatment is aimed at reducing ammonia levels through medication and dietary management [8,9]. The high mortality rate in UCDs is primarily due to the development of encephalopathy. This process is initiated by the following cascade of events: ammonia crossing the blood-brain barrier, its detoxification in astrocytes via the synthesis of glutamine from ammonia and glutamate, the breakdown of protective mechanisms and subsequent accumulation of glutamate, neurotoxicity mediated by excessive glutamate acting on N-methyl-D-aspartate (NMDA) receptors (an ionotropic glutamate receptor that selectively binds NMDA), ultimately leading to damage and death of nerve cells [3-9].

Consequently, there is currently a pressing need to expand newborn screening (NBS) panels using domestic test systems for the early detection of UCDs and the prompt initiation of specific treatment. This is crucial to reduce irreversible processes that lead to disability and increased mortality.

The aim of this study was to characterize a rare case of an inherited metabolic disease with neonatal manifestation that required hospitalization of the newborn in the Neonatal Intensive Care Unit (NICU) of the National Medical Research Center for Obstetrics, Gynecology, and Perinatology, named after Academician V.I. Kulakov.

## Case presentation

Patient history

The patient is a full-term newborn male, K., with a birth weight of 4120 g. He presented with symptoms of hyperammonemia that met the diagnostic criteria for an inherited metabolic disorder (as per the All-Russian Society of Pediatrics).

The diagnosis included an inherited metabolic disease - urea cycle disorder (ornithine transcarbamylase (OTC) deficiency) - accompanied by hyperammonemia, neonatal seizures, depression of central nervous system (CNS) functions, and disseminated intravascular coagulation (DIC) with gastric bleeding. The patient was also noted to be large for gestational age (LGA).

Neurosonography and abdominal as well as renal ultrasound examinations revealed no pathological findings.

Complete blood count showed no signs of inflammation; the systemic inflammatory response marker, C-reactive protein (CRP), was within the normal age range. Urinalysis showed no inflammatory changes. Biochemical blood analysis revealed elevated activity of the following enzymes: gamma-glutamyl transferase, creatine phosphokinase, and creatinine levels up to 11.7 µmol/L (In a full-term newborn, typical serum creatinine levels in the first few days of life are in the range of 35-90 µmol/L).

The patient is a full-term male infant born to a 38-year-old woman from her first pregnancy, which was conceived naturally. The mother's obstetric and gynecological history was significant for primary infertility (two years), external genital endometriosis, and uterine fibroids, which required surgical treatment (myomectomy without uterine cavity entry).

The mother had no significant chronic medical conditions. The first trimester of the current pregnancy was uneventful, and prenatal screening showed no abnormalities. The second half of the pregnancy was complicated by gestational diabetes mellitus (GDM), which required dietary management. Prior to delivery, ultrasonography and external measurements indicated a large-for-gestational-age fetus, leading to a planned cesarean delivery due to combined indications. The surgery and postpartum course were uneventful. The newborn's Apgar scores were eight at one minute and nine at five minutes, and no resuscitation measures were required. The infant was monitored in the well-baby nursery during the first day of life. He was put to the breast immediately after birth and was breastfed.

By the beginning of the second day of life, the infant developed respiratory distress and neurological signs of hyperexcitability, including irritability with a high-pitched cry, hypertonia, and exaggerated startle responses at 20. Consequently, the patient was transferred to the NICU for further observation, diagnostic evaluation, and treatment. Upon admission to the NICU, non-invasive respiratory support with Continuous Positive Airway Pressure (CPAP) was initiated. At 20 hours of life, his condition deteriorated precipitously. He exhibited a progressive decline in consciousness to a state of unresponsiveness to painful stimuli, accompanied by the development of generalized tonic seizures, characterized by sustained stiffening and extension of both upper and lower limbs. This clinical picture necessitated endotracheal intubation for airway protection and a transition to invasive mechanical ventilation (IMV). Throughout the observation period, he remained hemodynamically stable and did not require inotropic support. On the second day of life, the infant developed signs of coagulopathy, manifesting as “coffee-ground” gastric aspirates. This occurred despite intramuscular vitamin K prophylaxis at birth. Coagulation studies revealed a profile consistent with DIC: thrombocytopenia (platelets 85 × 10⁹/L), prolonged prothrombin time (28 seconds), prolonged activated partial thromboplastin time (65 seconds), and hypofibrinogenemia (1.1 g/L). This prompted discontinuation of enteral nutrition and administration of a fresh frozen plasma (FFP) transfusion. Instrumental investigations (ultrasound) revealed no structural pathology of the cardiovascular or digestive systems to account for the bleeding.

Additional history obtained from the mother revealed significant family history: one maternal aunt died at seven years of age from a metabolic disorder of unknown etiology, while another maternal aunt was found by exome sequencing to carry a pathogenic heterozygous mutation in the X-chromosomal OTC gene, associated with OTC deficiency and hyperammonemia, which leads to one of the forms of hyperammonemia.

Given the neurological status upon admission to the NICU (presence of neurological depression), significant hyperlactatemia on the second day of life (up to 9.4 mmol/L), and the progression of pathological neurological symptoms, the leading clinical suspicion was an inherited metabolic disorder. Crucially, the established family history of a confirmed pathogenic OTC mutation in a maternal aunt made OTC deficiency (OTCD) the primary diagnostic consideration. A consultation was immediately held with the Head of the Pediatrics Department.

Upon suspicion of a UCD, a classic emergency metabolic regimen was initiated. Enteral feeding was discontinued immediately. Intravenous fluid therapy was switched to a solution containing 10% dextrose at a high glucose infusion rate (GIR) of 8-10 mg/kg/minute to promote an anabolic state and suppress endogenous protein catabolism. All sources of parenteral amino acids and protein were discontinued. Blood glucose levels were monitored closely and remained within the normal range for age (4.5-6.0 mmol/L), with no episodes of hypoglycemia. Despite this intervention, venous ammonia levels continued to rise precipitously: 247 μmol/L upon admission, rising significantly to 622 μmol/L after 24 hours.

By the third day of life, the infant's condition deteriorated due to worsening pathological neurological symptoms, manifesting as progressive depression of CNS functions. Cerebral function monitoring (CFM) showed suppression of background bioelectrical activity with periodic epileptiform patterns, followed by the development of decorticate posturing (sustained tonic flexion of the upper and lower limbs) and persistent tongue fibrillations. Anticonvulsant therapy with intravenous diazepam boluses was administered for acute seizure control. Respiratory support (IMV) was maintained; the patient did not require supplemental oxygen and remained nil by mouth, with all nutrition and medications being provided intravenously. Blood gas analysis revealed worsening metabolic acidosis. Table 1 presents a diagram visually illustrating the dynamics of the infant's condition and the rapid progression of the disease during the Center stay.

**Table 1:** Dynamics of the child's condition Key: ↑ = Elevated, ↓ = Impaired/diminished.

Assessment Category	Day 1 General Care Ward	Day 2 NICU	Day 3 NICU
Clinical Status	Satisfactory (Enteral)	Severe (Parenteral)	Extremely Severe (Parenteral, Intubated)
Neurological Status	Age-Appropriate	↓ Depression Agitation	↓↓↓ Worsening Depression
Respiratory Support	Room Air	CPAP	Invasive Mechanical Ventilation
Ammonia (µmol/L) (Normal: 64-107)	↑ 247	↑↑ 622	↑↑↑ 1058
Lactate (mmol/L)	–	–	↑ 4.2-4.6
Outcome	–	–	Transfer to a Specialist Center

Given the distinctive family history, characteristic clinical presentation (development of progressive neurological symptoms in the infant at the beginning of the second day of life after an initial "honeymoon" period), and laboratory findings (rising hyperammonemia to 622 μmol/L in the absence of elevated inflammatory markers), the patient was diagnosed with: Inherited metabolic disorder: UCD, OTC deficiency? Hyperammonemia.

Due to the necessity of specific therapy aimed at reducing blood ammonia concentration, including peritoneal/hemodialysis, the infant was transferred to a specialized department (Department for Rare Diseases) of the Morozovskaya Children's City Clinical Hospital (GBUZ DZM Morozovskaya DKB). Despite maximal intensive care, including attempts at dialysis, the infant's condition was irreversible, and he died on the 10th day of life.

During follow-up, medical genetic counseling was performed, which confirmed the diagnosis of an inherited metabolic disorder. The boy's mother was also counseled by a geneticist at the stage of planning future pregnancies, and she was recommended to undergo medical genetic counseling.

## Discussion

This case provides a sobering and clinically instructive illustration of the lethal trajectory of neonatal-onset OTCD. The infant's clinical course was alarmingly prototypical, yet the extreme values and rapid progression offer critical insights. His plasma ammonia level skyrocketed to 1058 µmol/L by the third day of life, a concentration firmly associated with a grave prognosis and a high risk of mortality or severe neurological sequelae despite maximal intensive care [10]. This rapid biochemical deterioration, which compressed the characteristic "honeymoon period" into a mere 24-48 hours, underscores the aggressive phenotype of certain OTC mutations and the body's negligible capacity to handle an ammonia load in the context of a near-complete enzyme blockade. While the advanced capabilities of our tertiary center-specifically, specifically the rapid availability of ammonia testing and immediate critical care support, were pivotal in establishing the diagnosis and initiating stabilization, the tragic outcome starkly highlights the profound limitations of even the most swift postnatal response when confronting such extreme hyperammonemia. This experience stands in stark contrast to reports where intervention is triggered at significantly lower ammonia levels, a scenario often facilitated either by a known family history or, ideally, through the future implementation of expanded newborn screening (NBS) programs. For instance, studies by Summar et al. have demonstrated that the inclusion of UCDs in NBS via elevated glutamine or citrulline markers allows for pre-symptomatic diagnosis and intervention, which dramatically improves survival and neurodevelopmental outcomes compared to cases diagnosed after clinical presentation [11]. The temporal profile of our patient’s deterioration, from well-being to intubation within hours, serves as a powerful reminder that the window for effective intervention in neonatal OTCD is not merely critical but vanishingly narrow, and that public health strategies like NBS are crucial for changing this paradigm.

A critical and potentially modifiable factor in this tragic outcome was the missed opportunity for a proactive, prenatal risk assessment. The significant family history, comprising a maternal aunt who died at seven years of age from a suspected metabolic disorder and another maternal aunt identified as a carrier of a confirmed pathogenic OTC mutation, was not identified or acted upon during routine antenatal care. This failure in systematic and detailed family history gathering precluded the option of planning a delivery in a center colocated with immediate access to hemodialysis and a specialized metabolic genetics team, a key strategic step known to improve outcomes in anticipated cases of neonatal OTCD. As outlined by Simpson et al., a structured management plan for at-risk pregnancies, including prenatal diagnosis and coordinated delivery at a tertiary care facility, is the standard of care for families with a known history of UCDs, as it ensures the immediate initiation of life-saving measures and prevents the catabolic cascade that follows oral feeding [12]. This oversight underscores an urgent, systemic need to implement standardized, detailed genetic questionnaires in prenatal care protocols. These tools must explicitly and repeatedly inquire about consanguinity, unexplained infant or childhood deaths, and diagnosed neurological or metabolic conditions in the extended family, as a single affirmative answer can fundamentally alter perinatal management.

The systemic challenges exposed by this case, however, extend far beyond a single missed history. The mention of regional diagnostic delays is a central issue that warrants deeper analysis. While our tertiary center possessed the infrastructure for rapid ammonia testing, the subsequent journey to a definitive molecular diagnosis for this family, a process crucial for genetic counseling and prenatal planning, remains fraught with obstacles in our healthcare setting. This pathway often involves complex logistics, specialized laboratory referrals, and significant wait times, which are prohibitive in many non-specialized regions. To mitigate this critical gap, our center has begun advocating for and implementing an internal "metabolic emergency" protocol. This protocol mandates immediate stabilization, ammonia-level-guided management, and the parallel, expedited shipment of blood samples for targeted OTC gene sequencing upon first clinical suspicion, rather than awaiting further clinical deterioration or the results of other tests. This "diagnose and dispatch" approach aims to compress the diagnostic timeline dramatically. The development of high-throughput tandem mass spectrometry methods, as discussed by Burgard et al., has revolutionized NBS and offers a technological model for how rapid, multiplexed biomarker analysis could be adapted for rapid diagnostic confirmation in critical care settings, even in cases not captured by standard screening [13].

Furthermore, this case powerfully demonstrates that comprehensive genetic counseling must extend beyond the immediate parents to encompass the entire extended maternal family. The identification of a pathogenic OTC mutation in the maternal lineage transforms this from an individual tragedy into a pivotal event for preventive medicine. The mother's sisters, their daughters, and other female relatives are all at risk of being carriers, with associated reproductive risks for having affected male offspring. A proactive, family-system approach to counseling is therefore not merely an adjunct but an essential component of care, as it empowers at-risk relatives with the knowledge to make informed reproductive choices, such as preimplantation genetic diagnosis or prenatal testing in future pregnancies. The psychological and medical importance of this extended cascade screening is well-documented; Lichter-Konecki and colleagues emphasize that identifying female carriers in X-linked disorders like OTCD is fundamental for providing accurate recurrence risks and enabling reproductive options, thereby reducing the incidence and burden of the disease in the family lineage [14]. In conclusion, while the technical prowess of a tertiary center is vital for acute management, this case argues compellingly for a more robust, integrated approach that begins with meticulous prenatal screening, is supported by rapid in-house diagnostic pathways, and culminates in expansive family-wide genetic counseling to prevent the recurrence of such devastating disorders.

## Conclusions

This tragic case underscores that for neonatal OTCD, the window for effective intervention is not merely critical but vanishingly narrow, often closing before standard NBS results are available. Therefore, while expanding screening panels remains a vital public health goal, our experience unequivocally highlights the more immediate and life-dependent need for heightened clinical suspicion and the availability of rapid, point-of-care ammonia testing in any neonatal unit. The central, modifiable failure in this case was not a lack of tertiary-level intensive care, but a systemic deficit in prenatal genetic risk assessment. The profound lesson is that a detailed, multi-generational family history is a non-negotiable component of antenatal care. Had the significant maternal family history of a metabolic disorder and a confirmed OTC mutation been known, it would have triggered a completely different management pathway: delivery at a center equipped for immediate dialysis, pre-emptive diagnosis, and the avoidance of the catastrophic protein load that precipitated the hyperammonemic crisis. Consequently, the most crucial conclusion we draw is the imperative for exhaustive genetic counseling and cascade carrier testing for the entire maternal lineage following a diagnosis of OTCD. This transforms a single, devastating outcome into an opportunity for prevention, enabling informed reproductive choices and proper perinatal planning for future pregnancies within the family, thereby averting a recurrence of this preventable tragedy.
